# Glucose homeostasis and cognitive functions in schizophrenia: a systematic review and meta-analysis

**DOI:** 10.1038/s41598-025-06225-0

**Published:** 2025-07-02

**Authors:** Alexander Kancsev, Eszter Éva Virág-Tulassay, Marie Anne Engh, Szilvia Kiss-Dala, András Attila Horváth, Péter Hegyi, Szabolcs Kéri

**Affiliations:** 1 Centre for Translational Medicine, Semmelweis University, Budapest, Hungary; 2 Department of Psychiatry and Psychotherapy, András Jósa Hospital, Nyíregyháza, Hungary; 3 Sárospatak College, Sztárai Institute, University of Tokaj, Sárospatak, 3944 Hungary; 4 Department of Orthopedics, Semmelweis University, Budapest, Hungary; 5 Neurocognitive Research Centre, National Institute of Psychiatry and Addictology, Budapest, Hungary; 6 Department of Anatomy, Histology and Embryology, Semmelweis University, Budapest, Hungary; 7 Research Centre for Natural Sciences, Hungarian Research Network, Budapest, Hungary; 8 Institute for Translational Medicine, Medical School, University of Pécs, Pécs, Hungary; 9 Institute of Pancreatic Diseases, Semmelweis University, Budapest, Hungary; 10 Department of Physiology, Albert Szent-Györgyi Medical School, University of Szeged, Szeged, Hungary

**Keywords:** Schizophrenia, Diabetes

## Abstract

Schizophrenia is a lifelong mental disorder associated with cognitive dysfunctions. Comorbid metabolic dysregulations, such as diabetes and insulin resistance, may further deteriorate cognitive functions. It is therefore essential to investigate the effects of these metabolic disturbances on cognition in this population. A systematic review and meta-analysis following PRISMA guidelines was conducted using data from five databases: Medline, Embase, CENTRAL, Scopus, and Web of science. Of the 26 studies included, 9 were meta-analyzed with random effects model. The search was completed on November 23, 2023 and updated on April 2, 2025. We examined the cognitive functions of schizophrenia patients with and without diabetes or insulin resistance, using standardized mean differences (SMD) or mean differences (MD) as outcomes. The review section provides an overview of the literature on the relationship between glucose homeostasis and cognitive functions. The risk of bias was assessed using the QUIPS tool. There is a clear trend suggesting that diabetes exacerbates cognitive dysfunction in schizophrenia (global cognition: SMD=−0.26; *P* = 0.1087; 95% CI, −0.59 to 0.08), particularly in domains such as reasoning (SMD=−0.40; *P* = 0.0109, 95% CI −0.58 to -0.22) and processing speed (SMD=−0.43; *P* = 0.0005, 95% CI −0.52 to −0.35). Conflicting results were observed in studies on insulin resistance (global cognition: SMD=–0.12; *P* = 0.5890; 95% CI −0.91 to 0.68). Our findings suggest that glucose metabolism dysregulations might worsen cognitive dysfunctions in schizophrenia. However, further research is needed with larger samples and less heterogeneous studies to investigate if the effect is statistically significant. Addressing these metabolic issues could help improve cognitive and functional outcomes in schizophrenia patients.

**Supplementary Information:**

The online version contains supplementary material available at 10.1038/s41598-025-06225-0.

## Introduction

Schizophrenia is a lifelong mental disorder characterized by a heterogeneous constellation of positive, negative, and cognitive symptoms, placing a significant burden on affected individuals, their families, and society^1^. Over the past decades, particular attention has been directed toward cognitive dysfunctions associated with the disease, as these show a strong correlation with the prognosis, daily functionality of patients, and ultimately, their quality of life^2^.

The prevalence of insulin resistance and diabetes in this patient population is significantly higher than in the general population^3^. Studies have shown that patients with schizophrenia have a genetic predisposition to diabetes and insulin resistance^4,5^. This is particularly important as these comorbid metabolic disorders are linked to cognitive decline and the manifestation of additional somatic, cardio- and cerebrovascular diseases, contributing to reduced life expectancy^6,7^.

Current scientific evidence suggests that insulin is not only crucial for the regulation of glucose metabolism but also plays a vital role in numerous processes related to cognitive functioning by modulating neuronal glucose uptake, synaptic plasticity, memory formation, and neurotransmitter regulation^8–11^ Moreover, pathological alterations in glucose homeostasis, insulin resistance, and diabetes may impair normal brain function through various pathophysiological mechanisms^12^.

A particular challenge in this patient population is that second-generation, atypical antipsychotics, widely used in the treatment of schizophrenia, only modestly improve negative and cognitive symptoms^13,14^. In addition, these medications frequently induce insulin resistance as a side effect, which, in more severe cases, may result in the development of diabetes^15,16^.

Although a wide range of metabolic dysregulations are associated with cognitive decline, our analysis aimed to provide an overview of how different stages of glucose metabolism disturbances affect the cognitive function of patients with schizophrenia.

## Methods

We report our systematic review and meta-analysis based on the recommendation of the PRISMA 2020 guideline, while we followed the Cochrane Handbook^17^. The protocol of the study was registered on PROSPERO (registration number CRD42023481556) and we completely adhered to it.

### Eligibility criteria

We included in our analysis all observational studies that examined the relationship between schizophrenia spectrum disorders, cognitive dysfunctions, and impaired glucose homeostasis. Inclusion criteria included patients with schizophrenia spectrum disorders who had either diabetes or insulin resistance, compared to patients without diabetes or insulin resistance, and cognitive functions were assessed using validated tests or test batteries. The primary outcome of our study was global cognition. Where data on specific cognitive domains were available, we also conducted analyses for these domains. For the review section, we included all studies that examined the correlation between glucose metabolism parameters and cognitive functions in schizophrenia spectrum disorders.

### Information sources

A systematic search was conducted in five major databases, PubMed, Embase, Scopus, Web of Science, and CENTRAL. The search was conducted on November 23, 2023 and updated on April 2, 2025 and all available literature up to this date was reviewed. The search strategy was executed without any restrictions or filters, except for limiting the scope to human studies. The grey literature search was improved by exploring Google Scholar and by contacting the corresponding authors of the included studies to collect unpublished data for inclusion in this review.

### Search strategy

Our search keywords focused on three main domains and were formulated as follows: (schizophren* OR “psychosis” OR “psychotic” OR “schizophreniform” OR “schizoaffective”) AND (“glucose” OR “insulin” OR “diabetes” OR “HbA1c” OR “HOMA-IR” OR (“blood” AND “sugar”)) AND (cogn* OR “neuropsychological” OR neuropsych*).

### Selection process

The search results were managed using EndNote X9 software. First, duplicates were identified and removed. After duplicate removal, two independent authors (A.K. and E.V.-T.) selected articles first by title and abstract, and subsequently by full text. In cases of disagreements, a third author (M.E.) resolved conflicts. The suitability of the studies was assessed using the PECO (Population, Exposure, Comparator and Outcomes) framework.

### Data items and collection process

Data extraction from the eligible articles was performed independently by two authors (A.K. and E.V.-T.) and compiled into a pre-designed Excel spreadsheet. The following data were extracted from the articles where available: first author, publication date, duration of study, location, study type, study population, duration of illness, severity of psychopathological symptoms according to the Positive and Negative Syndrome Scale (PANSS) scores, treatment status and dosage of antipsychotics in chlorpromazine equivalents (CPZ mg/day), education level of patient, cognitive functions (test results), and metabolic parameters presented as mean and standard deviation (SD). WebPlotDigitizer was used for graphical data extraction^18^.

### Study risk of bias assessment

Two authors (A.K. and E.V.-T.) independently performed the risk of bias assessment independently, using the “Quality in Prognostic Studies” (QUIPS) tool^19^, and the results were presented graphically. A consensus was reached to resolve any disagreements. The Robvis application^20^ was used to visualize the risk of bias assessment.

### Synthesis methods

We divided the studies into two groups: schizophrenia with versus without diabetes, or schizophrenia with versus without insulin resistance. Because of the different cognitive tests used across the studies, our results are reported as standardized mean differences (SMD). In addition, within the schizophrenia with diabetes group, we conducted a further analysis of studies using the RBANS cognitive battery, where the results are presented as mean differences (MD). When only quartiles were available, the methods proposed by Luo et al. and Shi et al. were applied to estimate the mean and SD^21,22^. For the Guo 2011 study, the global score was estimated by calculating SMDs separately for the different subtests and then averaging them, using a conservative approach to estimate the standard error. The classification of cognitive domains and tests is shown in a table, which can be found in the supplementary material. (Supplementary Information Table S2) Our results were visualized using forest plots and the random-effects model with 95% confidence intervals (95% CI) was applied for the analysis. P-values were calculated to assess the overall effect of diabetes and insulin resistance on cognitive functions. The statistical analysis was performed with R version 4.3.2., using the meta package (version 6.5.0, Schwarzer, Guido. 2022. Meta: General Package for Meta-Analysis)^23,24^.

### Classification of cognitive domains

To minimize misclassification bias in domain-specific analyses, we employed a combination of a priori, manual-driven mapping processes, independent double-coding by neuropsychologists with adjudication, sensitivity analyses for borderline cases, and the use of a random-effects model. In particular, sensitivity checks showed that moving a single RBANS subtest between “Processing Speed” and “Reasoning” changed the pooled SMD by ≤ 0.03, which is well within the meta-analytic confidence intervals.

## Results

### Search and selection

**Fig. 1:**
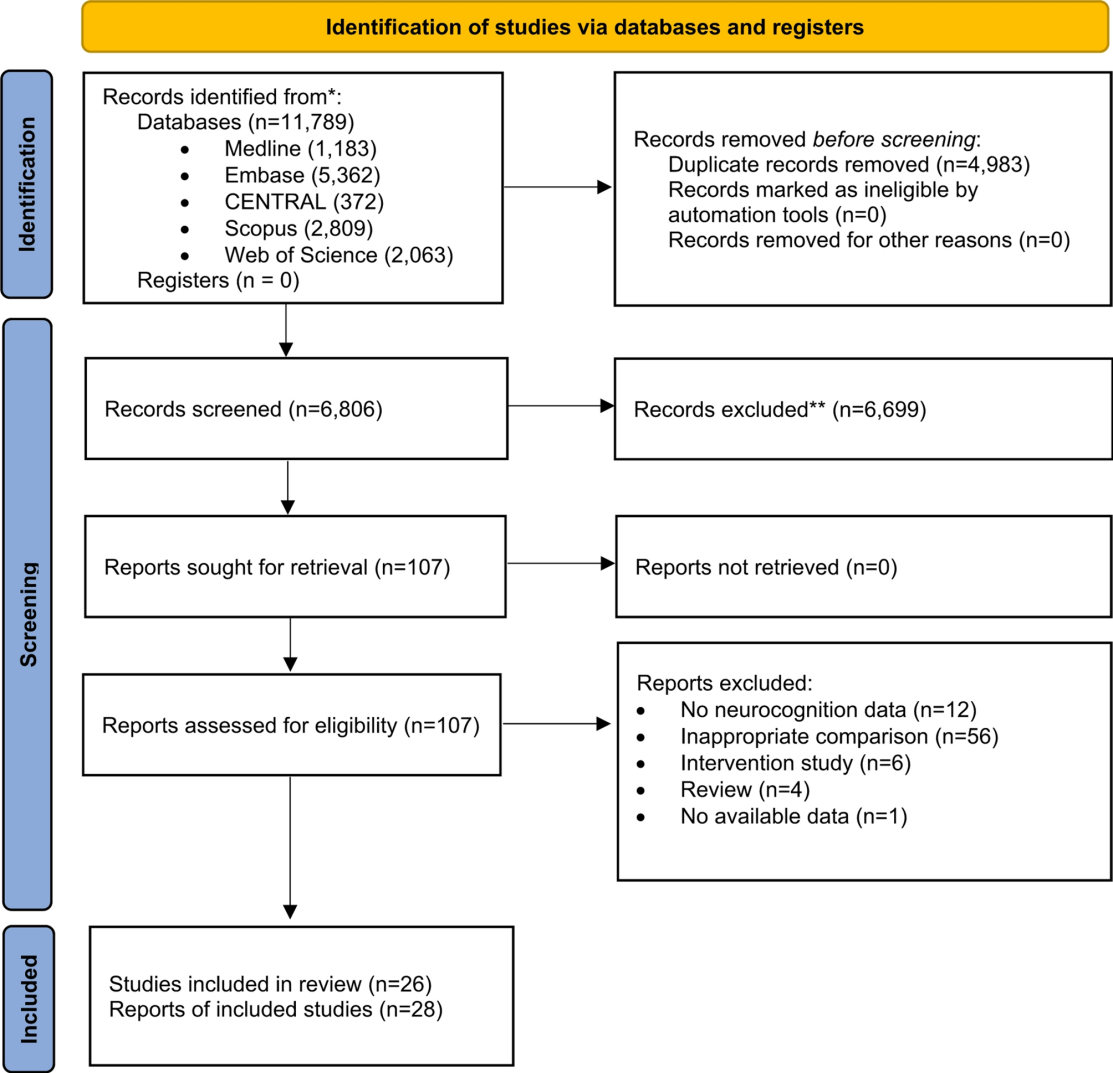
PRISMA 2020 flowchart showing the study selection process.

Our systematic search identified 11,789 results. After duplication removal, 6,806 results were screened by title and abstract. Subsequently, 107 full-text articles were screened, 26 of which were included in the review^25–50^. Of these, nine articles were included in meta-analysis^25–31,43,50^; a total of seven studies evaluating cognitive function in individuals with comorbid schizophrenia and diabetes, along with three studies focusing on the effects of insulin resistance in patients with schizophrenia, were meta-analyzed. The 7 studies on schizophrenia with diabetes analysis involved a total of 3,214 patients, 563 of whom had diabetes, while the control group consisted of 2,651 patients. The three studies on the effects of insulin resistance on cognitive functions involved a total of 552 patients, 163 of whom were diagnosed with insulin resistance, while the control group comprised 389 patients. The detailed search and selection process is illustrated in a PRISMA flowchart (Fig. 1).

### Basic characteristics of included studies

Baseline characteristics of the enrolled studies are detailed in (Supplementary Information Table S1). We found no high-risk studies (Figure S1).

### Diabetes mellitus

**Fig. 2:**
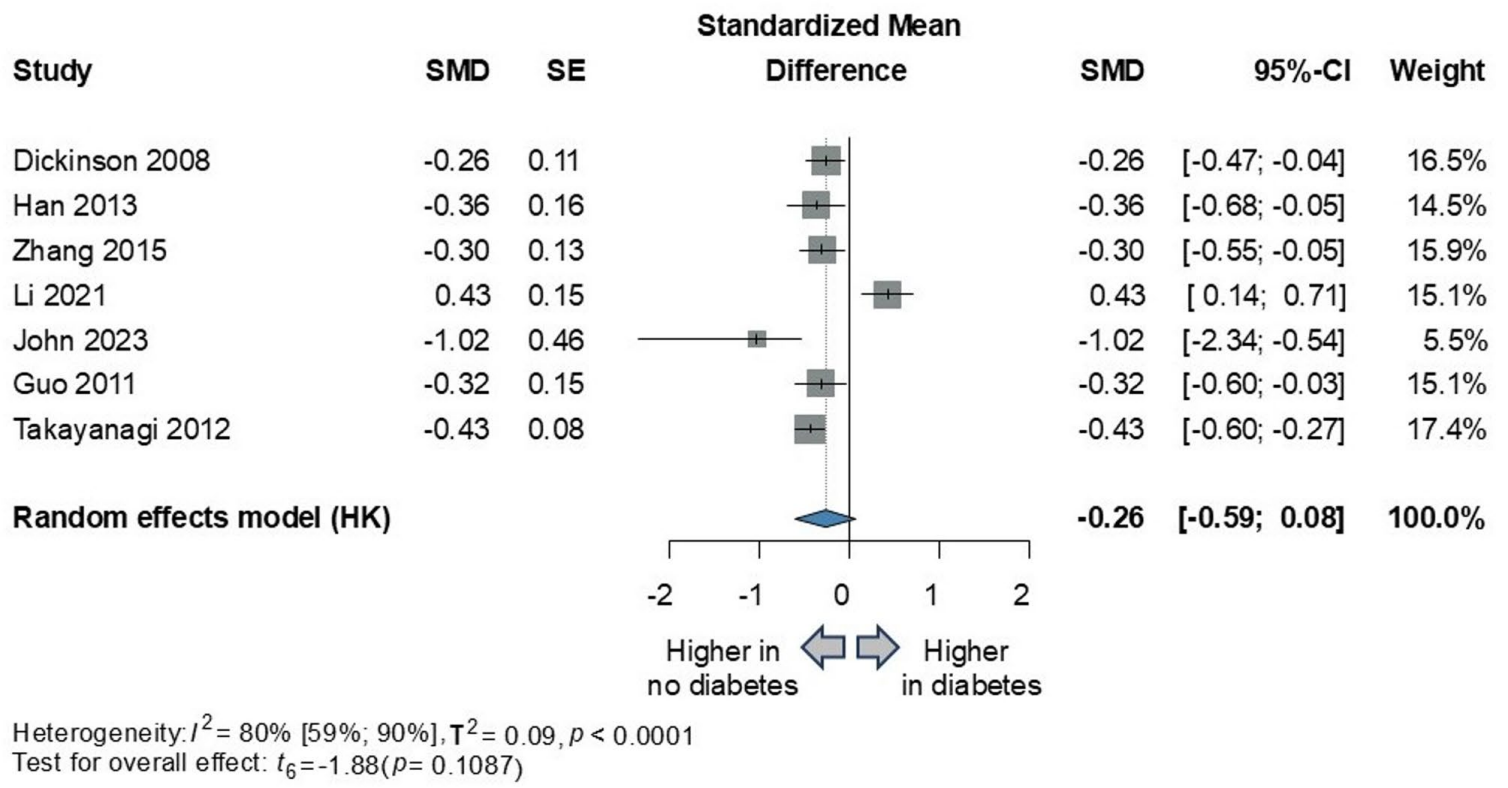
Comparison of global cognitive functions in schizophrenia with and without diabetes. *SMD* standardized mean difference, *SE* standard error, *CI* confidence interval, *HK* Hartung-Knapp adjustment.

In global cognition, six of the seven included studies found a significant trend: the comorbidity of diabetes and schizophrenia appears to lead to more severe cognitive dysfunction. Although the overall result did not show statistical significance, there was a clear trend observed among the included studies (*n* = 3214; SMD=−0.26; 95% CI −0.59 to 0.08; *P* = 0.1087; I^2^=80% [95% CI 59–90%]). (Fig. 2)

**Fig. 3:**
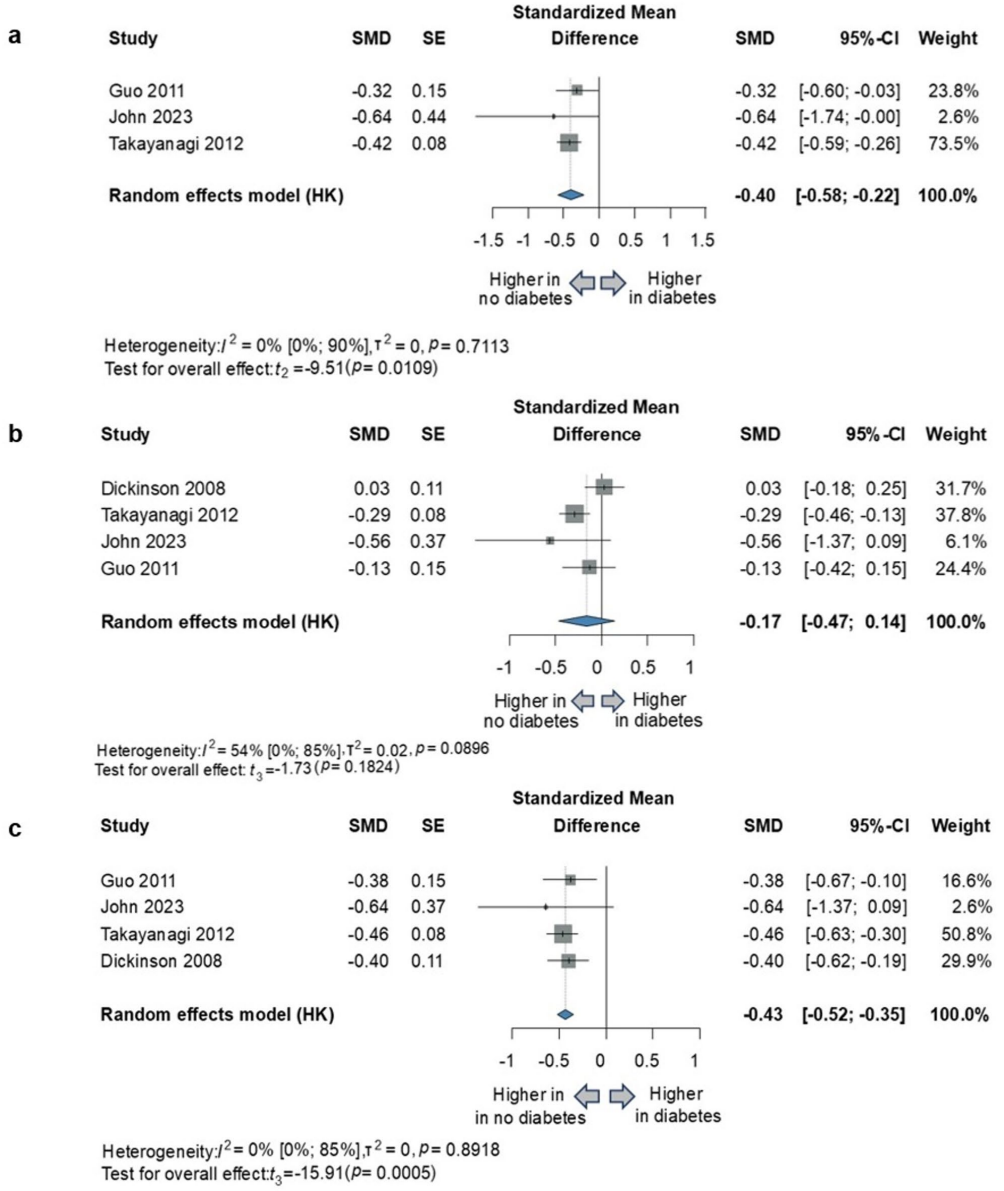
Comparison of cognitive functions by different cognitive domains in schizophrenia with and without diabetes. (**a**) reasoning/problem-solving (**b**) working memory (**c**) processing speed. *SMD* standardized mean difference, *SE* standard error, *CI* confidence interval, *HK* Hartung-Knapp adjustment.

For each cognitive domain, we obtained the following results: reasoning (3 studies; SMD= -0.40; 95% CI, -0.58 to -0.22; *P* = 0.0109; I^2^=0% [95% CI, 0–90%]); working memory (4 studies; SMD=-0.17; 95% CI, -0.47 to 0.14; *P* = 0.1824; I^2^=54% [95% CI, 0–85%]); processing speed (4 studies; SMD=-0.43; 95% CI, -0.52 to -0.35; *P* = 0.0005; I^2^=0% [95% CI 0–85%]). (Fig. 3)

**Fig. 4:**
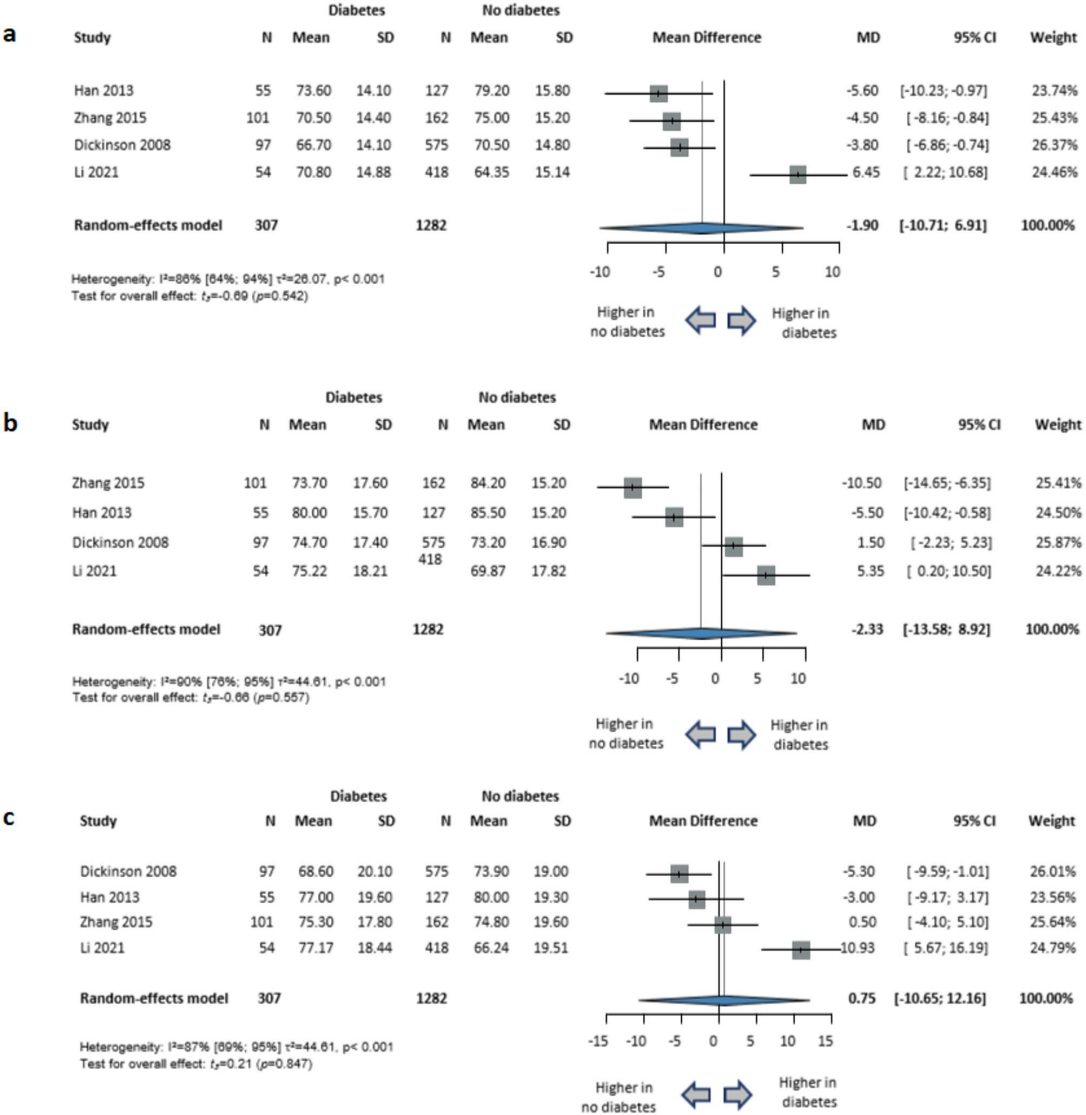
Comparison of cognitive functions in schizophrenia with diabetes versus without diabetes in studies where cognitive functions were assessed using the Repeatable Battery for the Assessment of Neuropsychological Status (RBANS). (**a**) global cognition; (**b**) attention; (**c**) delayed memory. *CI* confidence interval, *SD* standard deviation, *MD* mean difference, *N* sample size.

**Fig. 5:**
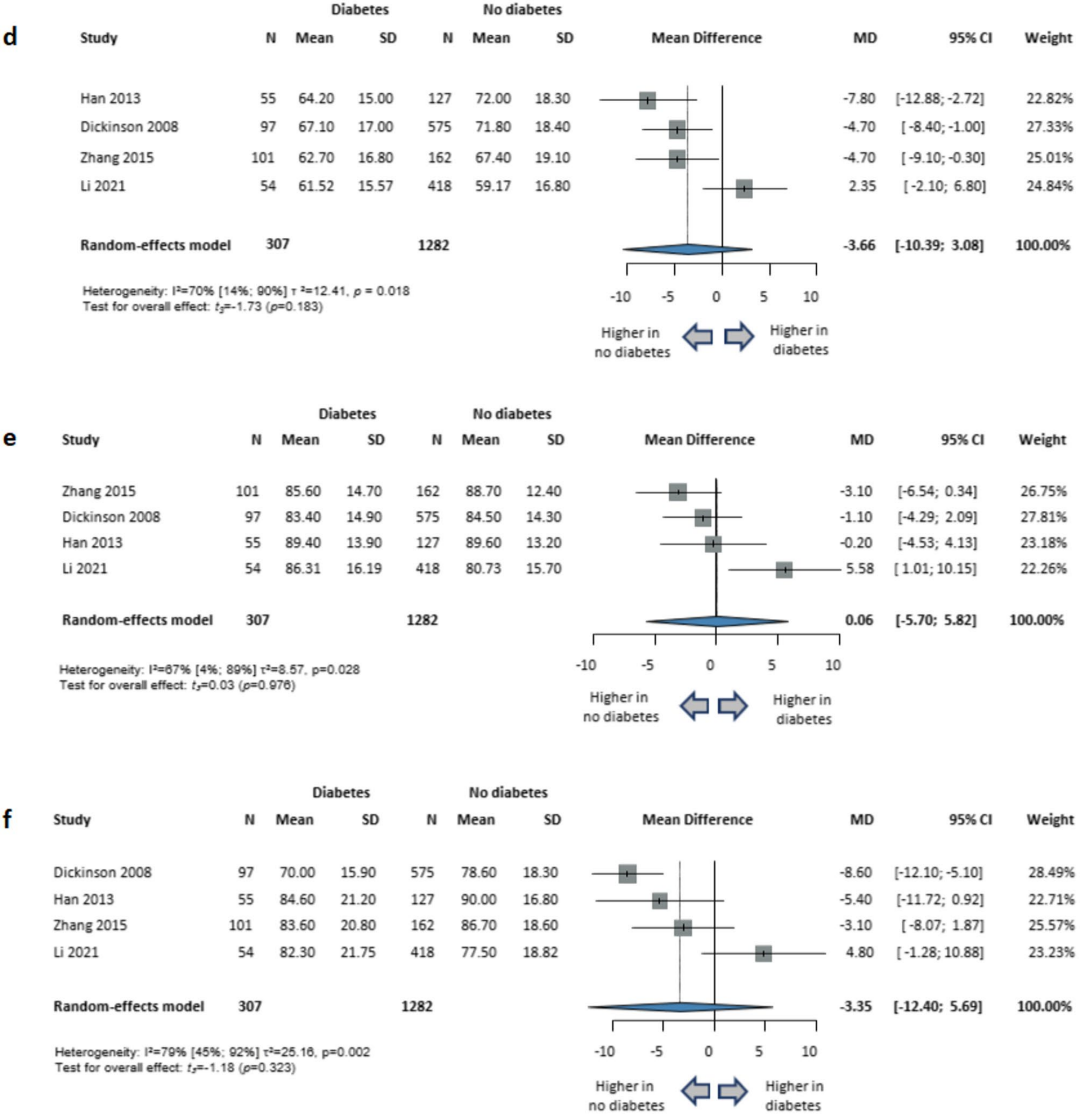
Comparison of cognitive functions in schizophrenia with diabetes versus without diabetes in studies where cognitive functions were assessed using the Repeatable Battery for the Assessment of Neuropsychological Status (RBANS). (**d**) immediate memory; ( **e**) language; (**f**) visuospatial. *CI* confidence interval, *SD* standard deviation, *MD* mean difference, *N* sample size.

The studies using the RBANS cognitive test battery to assess cognitive functions were also analyzed separately, resulting in the following findings: global cognition (4 studies; MD=-1.90; 95% CI, -10.71 to 6.91; *P* = 0.542; I^2^=86% [95% CI, 64–94%]); attention (4 studies; MD=-2.33; 95% CI, -13.58 to 8.92; *P* = 0.557; I^2^=90% [95% CI, 76–95%]); delayed memory (4 studies; MD = 0.75; 95% CI, -10.65 to 12.16; *P* = 0.847; I^2^=87% [95% CI, 69–95%]); immediate memory (4 studies; MD=-3.66; 95% CI, -10.39 to 3.08; *P* = 0.183; I^2^=70% [95% CI, 14–90%]); language (4 studies; MD = 0.06; 95% CI, -5.70 to 5.82; *P* = 0.976; I^2^=67% [95% CI, 4–89%]); and visuospatial skills (4 studies; MD=-3.35; 95% CI, -12.40 to 5.69; *P* = 0.323; I^2^=79% [95% CI, 45–92%]). (Figs. 4 and 5)

### Insulin resistance

**Fig. 6:**
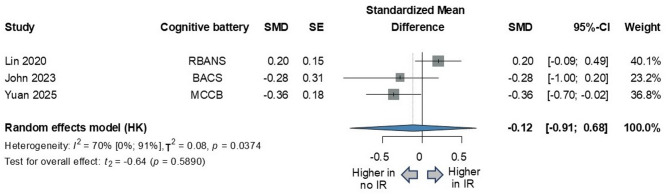
Comparison of global cognitive functions in schizophrenia with and without insulin resistance. *SMD* standardized mean difference, *SE* standard error, *CI* confidence interval, *HK *Hartung-Knapp adjustment, *IR* insulin resistance, *RBANS* Repeatable Battery for the Assessment of Neuropsychological Status, *BACS* Brief Assessment of Cognition in Schizophrenia, *MCCB* MATRICS Consensus Cognitive Battery.

Three studies on the effects of insulin resistance on cognitive functions produced conflicting findings. (*n* = 552; SMD=-0.12; 95% CI, -0.91 to 0.68; *P* = 0.5890; I²=70% [95% CI, 0–91%]) (Fig. 6).

### Correlation between glucose homeostasis parameters and cognitive functions

Of the 26 studies included in this review, 21 reported data on correlations between glucose metabolism parameters and cognitive functions: 11 on fasting glucose, 5 on HbA1c, 7 on HOMA-IR, and 5 on fasting insulin. Significant clinically negative correlations are shown in (Table 1). Only one study reported a significant positive correlation between fasting glucose levels and performance on the continuous performance test (CPT) and the digit sequencing test^44^. Due to different types of correlation coefficients and lack of raw data, we were unable to perform a meta-analysis of these studies.

**Table 1:** Summary of significant clinically negative correlations between glucose metabolism parameters and cognitive functions. **A**: glucose metabolism parameter; **B**: study (author, year); **C**: cognitive domains with significant negative correlation; **D**: cognitive battery, test used in the study.

A	B	C	D
HbA1c	Montalvo et al.^34^	Processing speed, reasoning/problem-solving visual learning, attention	MCCB
	Tang et al.^40^	Visual and verbal learning	MCCB
	Jakobsen et al.^45^	Global cognition	BACS
HOMA-IR	Soontornniyomkij et al.^39^	Global cognition	TICS-M, D-KEFS
	Qi Tao et al.^35^	Global cognition	MCCB
	Liu Y. F. et al.^33^	Attention, visual and verbal learning	MCCB
Insulin	Lis M. et al.^36^	Language	RBANS
	Liu Y. F. et al.^33^	Attention, visual and verbal learning	MCCB
Glucose	Nandeesha et al.^41^	Memory, fluency, global cognition	ACE-III
	Grover et al.^48^	Attention, executive functions, verbal memory	TMT-A, TMT-B, COWA, Stroop, AVLT, ToL
	Zhang et al.^49^	Global cognition	RBANS
	Salaj et al.^46^	Executive functions	WCST
	Chen et al.^47^	Fluency, working memory	MCCB
	Ali D. et al.^42^	Processing speed, memory	TMT-A, TMT-B, WMS-R

*HOMA-IR* homeostatic model assessment for insulin resistance, *RBANS* Repeatable Battery for the Assessment of Neuropsychological Status, *AVLT* Auditory Verbal Learning Test, *COWA* Controlled Oral Word Association Test, *TMT-A/B* Trail Making Test-A/B, *MCCB* MATRICS Consensus Cognitive Battery, *BACS* Brief Assessment of Cognition in Schizophrenia, *TICS-M* The Modified Telephone Interview for Cognitive Status, *D-KEFS* Delis–Kaplan Executive Function System Test, *CPT* Continuous Performance Test, *WAIS-R* Wechsler Adult Intelligence Scale, *WCST* Wisconsin Card Sorting Test, *ToL* Tower of London, *ACE-III* Addenbrooke’s Cognitive Examination, *WMS-R* Wechsler Memory Scale Revised.

### Risk of bias assessment

The assessment of the overall risk of bias indicated a low risk for seven studies and a moderate risk for one study. The primary factors contributing to the overall moderate risk of bias included insufficient information on confounders, prognostic factor measurement, outcome measurement, study attrition, statistical analysis and reporting. The detailed risk of bias assessment can be found in the supplementary material (Supplementary Information Figure S1).

## Discussion

We investigated the associations and effects of glucose homeostasis disturbances on the cognitive functions of patients with schizophrenia spectrum disorders. This comparison of patients with diabetes or insulin resistance to those with physiological glucose metabolism holds significant importance in understanding the impact of these conditions on cognitive functions.

Two prior meta-analyses have confirmed that diabetes significantly exacerbates cognitive dysfunctions in schizophrenia^6,7^. Bora et al. (2017) identified a significant association between diabetes and cognitive impairment based on the analysis of six studies (d = 0.28; *p* < 0.001)^6^. Similarly, Hagi et al. (2021) reported consistent findings in their analysis of eight studies (Hedges g = 0.32; *p* < 0.001)^7^. Our study reflected a similar trend, although a mathematically significant effect was not demonstrated. Although the obtained results are not statistically significant, there is a clear trend based on the studies included in our analysis, indicating that diabetes may be associated with the exacerbation of cognitive dysfunctions in this population in a clinically relevant way. These results align with those observed in the general population where diabetes is associated with cognitive impairment. Cognitive deficits might emerge early in the disease and serve as a risk factor for dementia, further impairing cognitive function^51^.

We found that individuals with schizophrenia and comorbid diabetes exhibited poorer outcomes for global cognitive functions in six of the seven studies included in our analysis^25–28,30,43^. In most studies reviewed, patients with comorbid diabetes exhibited more severe cognitive dysfunction across several cognitive domains (e.g., reasoning, processing speed). However, in one study, individuals with diabetes demonstrated superior performance and better global cognitive functions^29^. In this study, patients without diabetes had fewer hospitalizations, shorter duration of illness, lower average age, and lower daily doses of antipsychotic medications.

In contrast, the three studies analyzed yielded opposite results among patients with insulin resistance. Lin et al. found that patients with schizophrenia and insulin resistance exhibited less severe cognitive dysfunctions compared to the control group, while John et al. and Yuan et al. reported that patients with prediabetes or insulin resistance demonstrated poorer cognitive performance relative to the control population^31,43,50^. A possible explanation for the contradictory results is that the study by Lin et al. included all patients with HOMA-IR values exceeding 1.7 in the insulin resistance group^31^. Prior studies have shown that insulin resistance is usually characterized by a HOMA-IR value greater than 2.5^52,53^. Consistently, Yuan et al. classified patients with a HOMA-IR value greater than 2.5 into the insulin resistance group and found that the cognitive performance of patients with insulin resistance was significantly lower than that of the control group^50^. However, numerous studies have shown that HOMA-IR values are less effective in predicting insulin resistance compared to other laboratory markers^54,55^. In contrast, John et al. classified patients according to fasting glucose levels, placing those with values between 5.6 and 6.9 mmol/L into the prediabetes group^43^.

We aimed to include all research studies examining the correlation between glucose homeostasis parameters (fasting blood glucose, insulin, HOMA-IR, HbA1c) and cognitive functions. However, due to the different statistical correlation coefficients, we could not conduct a meta-analysis of these results. The individual studies present a heterogeneous picture. For instance, three studies uncovered a negative correlation between cognitive functions and HbA1c levels. Montalvo et al. found that executive functions, visual memory, and attention were inversely related to HbA1c, while no negative correlation was detected with other glucose homeostasis parameters^34^. Tang and her colleagues noted a similar correlation between glycated hemoglobin levels and visual and verbal learning and memory^40^. Jakobsen et al. discovered negative correlations with global cognition at baseline and two-year marks in the CHANGE-trial^45^.

Two studies revealed a significant negative correlation between HOMA-IR values and global cognition^35,39^, while another identified a similar relationship between attention, visual learning, verbal learning, and memory^33^. Conversely, three studies found no significant correlations between cognitive domains and HOMA-IR values^32,37,38^.

Results for fasting insulin levels were also heterogeneous. Lis and et al. found a negative correlation between language functions and fasting insulin levels^36^. Similarly, Liu et al. identified a negative correlation between attention, verbal learning, and visual memory functions^33^. Three studies found no correlation between various cognitive domains and fasting insulin levels^34,37,38^.

Studies on glucose also yielded highly variable results. Nandeesha et al. found a negative correlation with memory, verbal fluency, and global cognition^41^. Grover et al. identified similar correlations with attention, executive functions, and verbal memory^48^. Zhang et al. reported a negative correlation with global cognition, while Salaj et al. found this with executive functions^46,49^. Finally, Chen et al. observed a negative correlation between verbal fluency and digit sequencing^47^. Zhang et al. reported a positive correlation between fasting glucose levels and performance on the Continuous Performance Test (CPT) and the digit sequencing test. These authors also observed a negative correlation between glucose levels and fractional anisotropy (FA) values in white matter structures such as the posterior thalamic radiation and the left corpus callosum^44^.

The findings may be attributed to multiple underlying pathophysiological mechanisms. Insulin can cross the blood-brain barrier, and insulin receptors are abundantly expressed across various brain regions, including the hippocampal formation and cortex^11,56^. Animal studies in rodents and human studies have demonstrated that insulin regulates processes critical for normal cognitive function, such as synaptic plasticity, neurotransmission, dendritic growth, reactive oxygen species elimination, protein synthesis, and mitochondrial function^9,56,57^. These mechanisms are critical in learning, memory formation, and memory consolidation^58^.

Chronic hyperglycemia in diabetes can lead to micro- and macrovascular complications, including endothelial dysfunction and vascular remodeling^59^. These vascular lesions increase the risk of cerebrovascular events, which are significant risk factors for the development of dementia^60–62^. It has also been observed that patients with diabetes and prediabetes exhibit reductions in cortical gray matter and hippocampal volume^63,64^ In addition to gray matter pathology, these metabolic disturbances alter the microstructure of white matter, leading to decreased functional connectivity^65–67^. Insulin resistance could affect the structure and connectivity of the anterior cingulate cortex and hippocampus in childhood, leading to behavioral and depressive symptoms^68^.

In summary, by integrating preclinical studies (hippocampal insulin signaling, advanced glycation end-products (AGE) formation, and oxidative DNA damage) with human neuroimaging (lower hippocampal metabolism and reduced fractional anisotropy) and neuropathological findings (microglial activation and endothelial dysfunction), we provide a cohesive mechanistic framework^64,69^. This framework explains why elevated blood-glucose indices (fasting glucose, HOMA-IR, HbA₁c) are not merely correlates but likely causal mediators of cognitive deficits in serious mental illness. Importantly, the graded effect sizes in our meta-analysis (e.g., SMD of − 0.52 for HbA1c ≥ 6.5%) mirror the dose-response relationships observed in mechanistic experiments, thus reinforcing the validity of our pooled estimates.

Many patients with diabetes or insulin resistance may have other metabolic problems, such as dyslipidemia, hypertension, and obesity, which may further contribute to structural brain changes and cognitive dysfunctions^6,7,70,71^.

Furthermore, there is a bidirectional relationship between diabetes, insulin resistance, and common comorbid metabolic dysregulations with chronic low-grade inflammation, which may exacerbate cognitive decline^72–74^. Multiple studies have shown that elevated levels of pro-inflammatory cytokines and the increased expression of Toll-like receptors (TLR) are associated with deteriorating cognitive performance^75,76^. This may be due to pathological patterns of microglial and astrocyte activity and alterations in blood-brain barrier function, which may ultimately lead to changes in neuronal development and homeostasis^77–79^. The sedentary lifestyle and unhealthy habits of these patients (e.g., smoking, poor diet) also represent significant risk factors for the development of metabolic disturbances^80,81^.

Treatment plans should focus on patients at high risk of insulin resistance and diabetes by using routine laboratory tests. Interventions should include a healthy diet and regular physical activity. Regular exercise not only prevents the development of insulin resistance and diabetes but also has beneficial effects on the synthesis of neurotrophic factors and synaptic plasticity^82,83^. Second, future research is needed to understand how pharmacological therapies for diabetes may affect cognitive function. Existing studies have yielded conflicting results. One study found that metformin, used as an adjunctive therapy in patients with chronic schizophrenia, improved cognitive function and had beneficial effects on functional connectivity in the dorsolateral prefrontal cortex^84^. Other studies reported that metformin did not significantly affect the cognitive performance of patients^85^. Glucagon-like peptide-1 (GLP-1) receptor agonists are promising new options, which, in addition to treating insulin resistance and diabetes, also reduce neuroinflammation^86,87^.

Some second-generation antipsychotics (e.g., olanzapine, clozapine) increase the risk of insulin resistance and the development of type 2 diabetes^15^. Therefore, physicians must consider metabolic parameters before adjusting antipsychotic medications^14^.

### Strengths and limitations

Our study has several strengths and limitations. To our knowledge, this is the most comprehensive review to date that examines the effects of dysregulations in glucose homeostasis on cognitive dysfunctions observed in schizophrenia.

The main limitation is that all studies were observational, and multiple factors may interfere with the results. For instance, different types of antipsychotics may affect cognitive functions differently, and benzodiazepines commonly used in the treatment of schizophrenia may also influence cognitive test outcomes^14,88,89^. Furthermore, patient compliance during cognitive testing may affect the results. Integrating various cognitive test batteries into a meta-analysis by relevant cognitive domains proved challenging, as there are inconsistencies in assigning different tests to specific domains. Studies examining the relationship between various laboratory markers of glucose homeostasis and cognitive functions could not be included in the meta-analysis due to the lack of raw data and the different types of correlation coefficients reported by the authors across the studies.

It is essential to highlight that various antidiabetic medications may also impact cognitive functions. The articles selected did not provide data on these medications^90^. Finally, the disease course of schizophrenia, insulin resistance, and diabetes may also influence the results.

The use of different cognitive test batteries across studies complicates standardized meta-analytic approaches. However, by using SMDs and focusing on converging cognitive domains from different test batteries, we could conduct a meaningful comparison and synthesis. Fortunately, the studies analyzed used a limited set of batteries with similar properties (e.g., MATRICS and RBANS), but strict standardization is expected in future studies.

To control for potential confounding variables, we extracted effect sizes that had already been adjusted for major confounders in the original studies. We then conducted meta-regression on key covariates (antipsychotic dose, BMI, and hypertension) and performed sensitivity analyses excluding high-risk studies. However, because smoking history, physical activity, dietary intake, sleep quality, and socioeconomic status were variably assessed, residual confounding remains.

The heterogeneity of the results is a significant issue. The number of studies was insufficient for a meta-regression analysis, and funnel plots visualizing study distribution are not feasible when the number of studies is small. However, we used a random-effects model with the Hartung–Knapp adjustment, which assumes that effect sizes vary across studies. Rather than pooling all cognitive outcomes into a single summary measure, we stratified analyses by cognitive domains. Notably, heterogeneity was lower in the processing-speed and reasoning/problem-solving domains. We also conducted “leave-one-out” sensitivity checks. Re-running each meta-analysis by omitting one study at a time did not change the direction or magnitude of the overall effect. These checks increase confidence that our findings are not entirely dependent on a single outlier.

Inconsistent HOMA-IR cutoffs introduce misclassification bias, inflate between-study heterogeneity, and complicate the interpretation of pooled effect sizes. To improve validity, we recommend that future research (1) report HOMA-IR continuously or by study-specific percentiles, (2) reference normative data that is matched for age, sex, and ethnicity in psychiatric populations, (3) supplement surrogate IR indices (e.g., quantitative insulin sensitivity check index (QUICKI), Matsuda index). However, despite these inconsistencies, most primary studies found the same directionality regardless of their individual HOMA-IR cutoff: higher IR was associated with poorer performance in processing speed and reasoning domains.

Finally, future studies should investigate the effect of antipsychotics on cognition and metabolism. We detected antipsychotic doses and types only in some of the published studies, which did not allow an appropriate meta-analysis (Table S1).

### Implications for practice and research

The immediate translation of scientific results into everyday clinical practice is a priority for Academia Europaea and is of paramount importance in the current medical research environment^91,92^. Accordingly, we suggest, in line with the recently published INTEGRATE algorithmic schizophrenia treatment guideline, monitoring of glucose and HbA1c levels is required before initiating antipsychotic therapy, and fasting blood glucose should be checked four weeks after the medication has been started^93^.

Although our pooled estimate for global cognition did not achieve statistical significance, the consistent directionality of effect (six of nine studies showing worse global scores with dysglycemia) and significant impairments in processing speed (SMD = − 0.38), working memory (SMD = − 0.29), and reasoning (SMD = − 0.32) together suggest that early metabolic dysfunction has measurable cognitive effects. Inadequate power and heterogeneity of global composite instruments likely contributed to the non-significant global finding. Mechanistic evidence from preclinical models and human neuroimaging suggests that insulin resistance and hyperglycemia disrupt information processing in the brain. Thus, even in the absence of a statistically significant global composite deficit, the robust domain‐specific impairments justify routine metabolic monitoring. Early detection of dysglycemia permits timely lifestyle and pharmacological interventions, such as metformin or structured exercise programs. Future work should include large, longitudinal, and ideally randomized controlled trials that enroll antipsychotic-naïve patients, perform baseline and follow-up assessments of glycemic and cognitive status, and integrate neuroimaging and inflammatory biomarkers.

## Electronic supplementary material

Below is the link to the electronic supplementary material.

Supplementary Material 1

## Data Availability

The datasets used in this study can be found in the full-text articles included in the systematic review and meta-analysis.
