# Supplementary material for: Glucose homeostasis and cognitive functions in schizophrenia: a systematic review and meta-analysis
Source: Sci Rep. 2025 Jul 2;15:22898. doi: 10.1038/s41598-025-06225-0 (PMC12215534; doi:10.1038/s41598-025-06225-0)
Supplement: Supplementary file 1 — Supplementary Material 1 [file 41598_2025_6225_MOESM1_ESM.docx]

**Glucose homeostasis and cognitive functions in schizophrenia: a systematic review and meta-analysis**

**Authors**

Alexander Kancsev^1,2,3^, Eszter Éva Virág-Tulassay^1,4^, Marie Anne Engh^1^, Szilvia Kiss-Dala^1^, András Attila Horváth^1,5,6,7^, Péter Hegyi^1,8,9^, Szabolcs Kéri^1,3,10^

**Affiliations:**

1. Centre for Translational Medicine, Semmelweis University, Budapest, Hungary
2. Department of Psychiatry and Psychotherapy, András Jósa Hospital, Nyíregyháza, Hungary
3. Sztárai Institute, Sárospatak College, University of Tokaj, Sárospatak, Hungary
4. Department of Orthopedics, Semmelweis University, Budapest, Hungary
5. Neurocognitive Research Centre, National Institute of Psychiatry and Addictology, Budapest, Hungary
6. Department of Anatomy, Histology and Embryology, Semmelweis University, Budapest, Hungary
7. Research Centre for Natural Sciences, Hungarian Research Network, Budapest, Hungary
8. Institute for Translational Medicine, Medical School, University of Pécs, Pécs, Hungary
9. Institute of Pancreatic Diseases, Semmelweis University, Budapest, Hungary
10. Department of Physiology, Albert Szent-Györgyi Medical School, University of Szeged, Szeged, Hungary

**Corresponding author**

Szabolcs Kéri MD, PhD, DSc

Postal address: Sztárai Institute, University of Tokaj, 3944 Sárospatak, Hungary

E-mail address: [keri.szabolcs@unithe.hu](mailto:keri.szabolcs@unithe.hu)

***Table S1.*** *Basic characteristics of studies included*

|  | **Author (year)** | **Study site** | **Study design** | **Number of analyzed patients**  **Exposed**  **(female %)** | **Number of analyzed patients**  **Unexposed**  **(female %)** | **Total number of analyzed patients** | **Age (year) mean (SD)**  **Exposed/**  **unexposed** | **Exposed patients**  **(exposure type) % of total** | **PANSS**  **Mean/SD**  **Exposed** | **Educational**  **Years**  **Exposed/unexposed**  **Mean (SD)** | **Duration of illness (Y/M)**  **Mean/SD**  **Exposed** | **Cognitive battery** | **Treatment status (CPZ EQ.mg/Day)**  **Exposed**  **Mean/SD** |
| --- | --- | --- | --- | --- | --- | --- | --- | --- | --- | --- | --- | --- | --- |
| 1 | Dickinson et. al.  2008 | USA | cross-sectional | 97 (42) | 575 (32) | 672 | 48.1(8.9)/40.5(9.7) | 14.4 (D) | NA | NA | NA | RBANS | NA |
| 2 | Guo et. al. 2011 | China | cross-sectional | 78 (46.2) | 118 (44.1) | 196 | 44.6(7.6)/43.0(7.2) | 39.8 (D) | 63/12.8 | 12.5(2.7)/12.2(2.9) | 8.2/3.0(Y) | WAIS-R digit symbol, WAIS-R digit span TMT-A, TMT-B, WCST-128, WMS-R (visual reproduction) | NA |
| 3 | Han et. al. 2013 | China | case-control | 55 (38.2) | 127 (35.4) | 182 | 54.4(8.1)/53.3(8.3) | 30.22 (D) | 59.2/15.0 | 9.8(2.5)/9.7(2.4) | 11.6/9.3 (Y) | RBANS | 385.7/257.1 |
| 4.1 | Zhang et. al. 2015 | China | cross-sectional | 67 (0) | 125 (0) | 192 | 52.1(8.8)/50.9(8.1) | 34.9 (D) | 60.6/13.9 | 9.1(2.1)/9.7(2.4) | 27.0/9.8 (Y) | RBANS | 410.8/188.8 |
| 4.2 | Zhang et. al. 2015 | China | cross-sectional | 34 (100) | 37 (100) | 71 | 53.8(6.8)/51.2(6.4) | 47.89 (D) | 62.7/18.6 | 9.7(1.8)/9.9(2.4) | 25.2/9.6 (Y) | RBANS | 438.2/270.0 |
| 5 | Li et. Al. 2021 | China | cross-sectional | 54 (26) | 418 (11.2) | 472 | 52.72(8.31)/46.5(8.69) | 11.44 (D) | 57.87/12.98 | 9.35(2.6)/8.71(2.37) | 28.26/8.22 (Y) | RBANS | 589.50/782.09 |
| 6 | Takayanagi et. al.  2012 | USA | cross-sectional | 161 (31) | 1128 (24) | 1289 | 45.9(8.7)/39.6(11.1) | 12.49 (D) | 71.4/15.2 | 12.0(2.0)/12.2(2.2) | NA | controlled oral word, category instances, grooved pegboard, WAIS-R digit symbol, WCST-64, WISC-mazes, Hopkins verbal learning, comp. test of visuospatial working memory, letter-number seq, CPT, facial emotion discrimination test | NA |
| 7 | Lin et. al.  2020 | China | cross-sectional | 73 (39.73) | 120 (42.5) | 193 | 49.26(8.57)/51.05(6.8) | 37.82 (IR) | 75.60/14.72 | 11.15(2.73)/10.73(2.38) | 289.05/123.05 (M) | RBANS | 334.52/203.01 |
| 8 | Kowalski et. al. 2023 | Poland | cross-sectional | 100 (40) | 55 (45.5) | 155 | 43.7(12.0)/42.3(15.0) | 64.5 (SCH) | N: 16.0/9.1 | NA | 18.8/12.3 (Y) | RBANS | 608/373.5 |
| 9 | Liu et. al. 2018 | China | cross-sectional | 80 (NA) | 70 (NA) | 150 | NA | 53.3 (DN-SCH) | NA | NA | NA | MCCB | Drug naïve |
| 10 | Montalvo et. al. 2020 | Spain | cross-sectional | 60 (35) | 50 (44) | 110 | 24.5(5.4)/23.8(4.8) | 54.5 (SCH-SP) | NA | 11.3(2.8)/13.4(2.7) | NA | MCCB | 371.1/334.0 |
| 11 | Qi Tao et. al. 2020 | China | cross-sectional | 90 (51.1) | 70 (54.3) | 160 | 21.5(7.7)/23.4(5.4) | 56.3 (DN-SCH) | 84.2/12.7 | 10.4(2.6)/11.1(2.4) | 5.9/6.3 (M) | MCCB | Drug naive |
| 12 | Lis et. al. 2020 | Poland | cross-sectional | 35 (48.6) | 65 (64.6) | 100 | 34.2(12.5) | 35.0 (SCH-SP) | P:15/5.3, N:19.3/8.0 | 13.7(3.0) | NA | RBANS | 347.2/174.2 |
| 13 | Pang et. al. 2023 | China | cross-sectional | 142 (51) | 140 (49) | 282 | 25.0(4.0)/26.0(4.0) | 50.4 (DN-SCH) | 80.0/6.0 | 12.0(3.0) | 6.0/4.0 (M) | MCCB | Drug naive |
| 14 | Peng et. al. 2021 | China | cross-sectional | 172 (56) | - | 172 | 24.32(6.69) | 100 (DN-SCH) | 97.01/15.68 | 10.95(2.61) | 15.03/12.68 (M) | MCCB | Drug naive |
| 15 | Soontornniyomkij et. al. 2019 | USA | cross-sectional | 145 (46) | 140 (54) | 285 | 48.3(10.1)/48.7(11.2) | 50.9 (SCH) | NA | 12.4(2.3)/14.5(2.3) | 25.0/11.1 (Y) | TICS-M, D-KEFS | NA |
| 16 | Tang et. al. 2022 | USA | cross-sectional | 245 (33) | 165 (45) | 410 | 32.5(10.4)/32.0(10.4) | 59.8 (SCH) | NA | NA | NA | MCCB | NA |
| 17 | Nandeesha et. a. 2023 | India | cross-sectional | 200 (44) | 169 (36.1) | 369 | 35.66(9.44)/36.53(8.34) | 54.2 (DN-SCH) | 29.18/12.81 | NA | 3.5 (Y) | ACE-III | Drug naive |
| 18 | Ali et. al. 2020 | Egypt | cross-sectional | 40 (NA) | 20(NA) | 60 | NA | 66.7 (SCH) | NA | NA | NA | TMT-A, TMT-B, WMS-R | NA |
| 19.1 | John et. al. 2023 | Australia | cross-sectional | 17 (41.2) | 123(26.8) | 140 | 35.71(10.42)/31.51(10.4) | 12.14 (D) | NA | NA | 11.9/7.35 (Y) | BACS | NA |
| 19.2 | John et. al. 2023 | Australia | cross-sectional | 47 (36.2) | 123(26.8) | 170 | 34.43(9.66)/31.51(10.4) | 27.65 (IR) | NA | NA | 10.34/7.83 (Y) | BACS | NA |
| 20 | Zhang et. al. 2020 | China | cross-sectional | 39 (59) | 30 (56.7) | 69 | 26.5(6.3)/27.5(7.9) | 56.5 (DN-SCH) | NA | 12.4(3.1) | 23.3/25.1 (M) | MCCB | NA |
| 21 | Jakobsen et. al. 2018 | Denmark | cohort | 428 (55) | - | 428 | 38.6(12.4) | 100 (SCH-SP) | NA | NA | NA | BACS | 473.5/397.9 |
| 22 | Salaj et. al. 2014 | NA | NA | 27 (44) | - | 27 | 23.9(6.0) | 100 (DN-SCH) | NA | 9.6(2.8) | NA | WCST | Drug naive |
| 23 | Chen et. al. 2020 | China | cross-sectional | 158 (50.6) | - | 158 | NA | 100 (SCH) | NA | NA | NA | MCCB | NA |
| 24 | Grover et. al. 2019 | India | cross-sectional | 121 (45.5) | - | 121 | 33.89(9.86) | 100 (SCH) | 59.28/17.71 | 13.37(3.05) | NA | TMT-A, TMT-B, COWA, Stroop, AVLT, Tower of London | NA |
| 25 | Zhang et. al 2017 | China | cross-sectional | 216 (47) | - | 216 | 28.71(3.72) | 100 (SCH) | NA | 9.83(1.97) | NA | RBANS | NA |
| 26 | Yuan et. al. 2025 | China | cross-sectional | 43(58.1) | 146(56.8) | 189 | NA | 22.75 (IR) | 82.79/15.90 | NA | NA | MCCB | Drug naive |

***Abbreviations:*** *D: schizophrenia with diabetes; IR: schizophrenia with insulin resistance; SCH: schizophrenia; DN-SCH: drug naive or drug free schizophrenia; SCH-SP: schizophrenia spectrum; P/N: PANSS negative or positive subscore (when PANSS total score is unavailable); NA: not applicable; M: months; Y: years; RBANS: Repeatable Battery for the Assessment of Neuropsychological Status; AVLT: Auditory Verbal Learning Test; COWA: Controlled Oral Word Association Test; TMT-A/B: Trail Making Test-A/B; MCCB: MATRICS Consensus Cognitive Battery; BACS: The Brief Assessment of Cognition in Schizophrenia; WMS-R: The Wechsler Memory Scale; ACE-III: Addenbrooke's cognitive examination; TICS-M: The Modified Telephone Interview for Cognitive Status; D-KEFS: The Delis–Kaplan Executive Function System Test; CPT: Continuous Performance Test; WAIS-R: Wechsler Adult Intelligence Scale; WCST-64: Wisconsin Card Sorting Test-64; WISC: Wechsler Intelligence Scale for Children; WMS-R: The Wechsler Memory Scale-Revised*

***Table S2.*** *Classification of cognitive domains and tests included in the analysis*

| **Cognitive domains** | **Tests** |
| --- | --- |
| **Reasoning/**  **problem-solving** | - Wisconsin Card Sorting Test (WCST) - Tower of London (ToL) |
| **Working memory** | - Digit span - Computerized test of visuospatial working memory - Letter-Number Sequencing test (LNS) |
| **Speed of processing** | - Controlled Oral Word Association test (COWA) - Category instances - Grooved pegboard - Wecshler Adult Intelligence Scale-Revised (WAIS-R) - Digit symbol test - Trail Making Test A/B (TMT) - Verbal fluency - Symbol coding |

***Figure S1.*** *Risk of bias assessment across included studies*

***
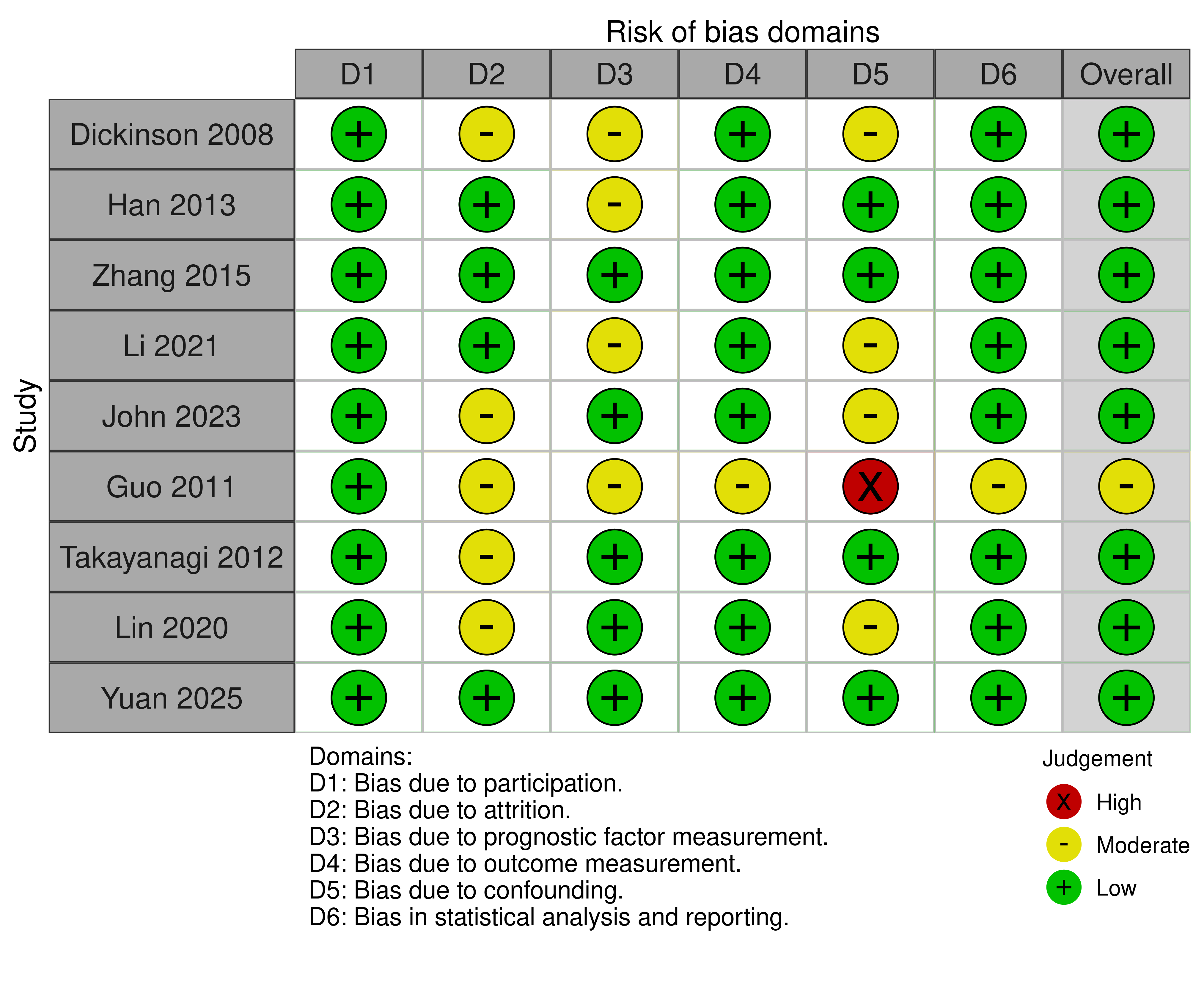
***

***Table S3***. *PRISMA 2020 Checklist*

| Section and Topic | Item # | Checklist item | Location where item is reported |
| --- | --- | --- | --- |
| **TITLE** | | |  |
| Title | 1 | Identify the report as a systematic review. | 1 |
|  | | |  |
| Abstract | 2 | See the PRISMA 2020 for Abstracts checklist. | 3 |
| **INTRODUCTION** | | |  |
| Rationale | 3 | Describe the rationale for the review in the context of existing knowledge. | 4 |
| Objectives | 4 | Provide an explicit statement of the objective(s) or question(s) the review addresses. | 4,5 |
| **METHODS** | | |  |
| Eligibility criteria | 5 | Specify the inclusion and exclusion criteria for the review and how studies were grouped for the syntheses. | 5 |
| Information sources | 6 | Specify all databases, registers, websites, organisations, reference lists and other sources searched or consulted to identify studies. Specify the date when each source was last searched or consulted. | 5 |
| Search strategy | 7 | Present the full search strategies for all databases, registers and websites, including any filters and limits used. | 5 |
| Selection process | 8 | Specify the methods used to decide whether a study met the inclusion criteria of the review, including how many reviewers screened each record and each report retrieved, whether they worked independently, and if applicable, details of automation tools used in the process. | 5,6 |
| Data collection process | 9 | Specify the methods used to collect data from reports, including how many reviewers collected data from each report, whether they worked independently, any processes for obtaining or confirming data from study investigators, and if applicable, details of automation tools used in the process. | 6 |
| Data items | 10a | List and define all outcomes for which data were sought. Specify whether all results that were compatible with each outcome domain in each study were sought (e.g. for all measures, time points, analyses), and if not, the methods used to decide which results to collect. | 6 |
|  | 10b | List and define all other variables for which data were sought (e.g. participant and intervention characteristics, funding sources). Describe any assumptions made about any missing or unclear information. | 6 |
| Study risk of bias assessment | 11 | Specify the methods used to assess risk of bias in the included studies, including details of the tool(s) used, how many reviewers assessed each study and whether they worked independently, and if applicable, details of automation tools used in the process. | 6 |
| Effect measures | 12 | Specify for each outcome the effect measure(s) (e.g. risk ratio, mean difference) used in the synthesis or presentation of results. | 6 |
| Synthesis methods | 13a | Describe the processes used to decide which studies were eligible for each synthesis (e.g. tabulating the study intervention characteristics and comparing against the planned groups for each synthesis (item #5)). | 5 |
|  | 13b | Describe any methods required to prepare the data for presentation or synthesis, such as handling of missing summary statistics, or data conversions. | 6 |
|  | 13c | Describe any methods used to tabulate or visually display results of individual studies and syntheses. | 6 |
|  | 13d | Describe any methods used to synthesize results and provide a rationale for the choice(s). If meta-analysis was performed, describe the model(s), method(s) to identify the presence and extent of statistical heterogeneity, and software package(s) used. | 6,7 |
|  | 13e | Describe any methods used to explore possible causes of heterogeneity among study results (e.g. subgroup analysis, meta-regression). | - |
|  | 13f | Describe any sensitivity analyses conducted to assess robustness of the synthesized results. | - |
| Reporting bias assessment | 14 | Describe any methods used to assess risk of bias due to missing results in a synthesis (arising from reporting biases). | 6 |
| Certainty assessment | 15 | Describe any methods used to assess certainty (or confidence) in the body of evidence for an outcome. | - |
| **RESULTS** | | |  |
| Study selection | 16a | Describe the results of the search and selection process, from the number of records identified in the search to the number of studies included in the review, ideally using a flow diagram. | 8,9 |
|  | 16b | Cite studies that might appear to meet the inclusion criteria, but which were excluded, and explain why they were excluded. | 9 |
| Study characteristics | 17 | Cite each included study and present its characteristics. | Table S1 |
| Risk of bias in studies | 18 | Present assessments of risk of bias for each included study. | 16, Fig S1 |
| Results of individual studies | 19 | For all outcomes, present, for each study: (a) summary statistics for each group (where appropriate) and (b) an effect estimate and its precision (e.g. confidence/credible interval), ideally using structured tables or plots. | 10-16 |
| Results of syntheses | 20a | For each synthesis, briefly summarise the characteristics and risk of bias among contributing studies. | Table S1, Fig S1 |
|  | 20b | Present results of all statistical syntheses conducted. If meta-analysis was done, present for each the summary estimate and its precision (e.g. confidence/credible interval) and measures of statistical heterogeneity. If comparing groups, describe the direction of the effect. | 10-15 |
|  | 20c | Present results of all investigations of possible causes of heterogeneity among study results. | - |
|  | 20d | Present results of all sensitivity analyses conducted to assess the robustness of the synthesized results. | - |
| Reporting biases | 21 | Present assessments of risk of bias due to missing results (arising from reporting biases) for each synthesis assessed. | Fig S1 |
| Certainty of evidence | 22 | Present assessments of certainty (or confidence) in the body of evidence for each outcome assessed. | - |
| **DISCUSSION** | | |  |
| Discussion | 23a | Provide a general interpretation of the results in the context of other evidence. | 17-22 |
|  | 23b | Discuss any limitations of the evidence included in the review. | 20-21 |
|  | 23c | Discuss any limitations of the review processes used. | 20-21 |
|  | 23d | Discuss implications of the results for practice, policy, and future research. | 21-22 |
| **OTHER INFORMATION** | | |  |
| Registration and protocol | 24a | Provide registration information for the review, including register name and registration number, or state that the review was not registered. | 5 |
|  | 24b | Indicate where the review protocol can be accessed, or state that a protocol was not prepared. | 5 |
|  | 24c | Describe and explain any amendments to information provided at registration or in the protocol. | 5 |
| Support | 25 | Describe sources of financial or non-financial support for the review, and the role of the funders or sponsors in the review. | 2 |
| Competing interests | 26 | Declare any competing interests of review authors. | 2 |
| Availability of data, code and other materials | 27 | Report which of the following are publicly available and where they can be found: template data collection forms; data extracted from included studies; data used for all analyses; analytic code; any other materials used in the review. | 2 |

*From:*  Page MJ, McKenzie JE, Bossuyt PM, Boutron I, Hoffmann TC, Mulrow CD, et al. The PRISMA 2020 statement: an updated guideline for reporting systematic reviews. BMJ 2021;372:n71. doi: 10.1136/bmj.n71. This work is licensed under CC BY 4.0. To view a copy of this license, visit https://creativecommons.org/licenses/by/4.0

**REFERENCES**

Ali, D. H. *et al.* Schizophrenic patients’ cognitive functions in relation to their metabolic profile: a cross-sectional, comparative study on an Egyptian sample. *Middle East Current Psychiatry* **27**, 46 https://doi.org/10.1186/s43045-020-00053-w (2020).

Chen, S. *et al.* The correlation between metabolic syndrome and neurocognitive and social cognitive performance of patients with schizophrenia. *Psychiatry Res* **288**, 112941 doi:10.1016/j.psychres.2020.112941 (2020).

Dickinson, D., Gold, J. M., Dickerson, F. B., Medoff, D. & Dixon, L. B. Evidence of exacerbated cognitive deficits in schizophrenia patients with comorbid diabetes. *Psychosomatics* **49**, 123–131 doi:10.1176/appi.psy.49.2.123 (2008).

Grover, S. *et al.* Relationship of metabolic syndrome and neurocognitive deficits in patients with schizophrenia. *Psychiatry Res* **278**, 56–64 doi:10.1016/j.psychres.2019.05.023 (2019).

Guo, X. *et al.* Cognitive functioning in schizophrenia with or without diabetes. *Zhong Nan Da Xue Xue Bao Yi Xue Ban* **36**, 724–727 doi:10.3969/j.issn.1672-7347.2011.08.004 (2011).

Han, M. *et al.* Diabetes and cognitive deficits in chronic schizophrenia: a case-control study. *PLoS One* **8**, e66299 doi:10.1371/journal.pone.0066299 (2013).

John, A. P., Mya, T. & Haywood, D. Cognitive deficits among people with schizophrenia and prediabetes or diabetes. *Acta Psychiatr Scand* **149**, 65–76 doi:10.1111/acps.13627 (2024).

Kowalski, K. *et al.* Altered levels of fecal short-chain fatty acids are associated with subclinical inflammation and worse cognitive performance in patients with schizophrenia. *J Psychiatr Res* **165**, 298–304 doi:10.1016/j.jpsychires.2023.07.042 (2023).

Li, S., Chen, D., Xiu, M., Li, J. & Zhang, X. Y. Diabetes mellitus, cognitive deficits and serum BDNF levels in chronic patients with schizophrenia: A case-control study. *J Psychiatr Res* **134**, 39–47 doi:10.1016/j.jpsychires.2020.12.035 (2021).

Lin, C. *et al.* The prevalence, risk factors, and clinical characteristics of insulin resistance in Chinese patients with schizophrenia. *Compr Psychiatry* **96**, 152145 doi:10.1016/j.comppsych.2019.152145 (2020).

Lis, M. *et al.* Assessment of Appetite-Regulating Hormones Provides Further Evidence of Altered Adipoinsular Axis in Early Psychosis. *Front Psychiatry* **11**, 480 doi:10.3389/fpsyt.2020.00480 (2020).

Liu, Y. F. *et al.* [Correlation of serum level of homocysteine and insulin resistance with cognitive dysfunction in first-episode schizophrenics]. *Zhonghua Yi Xue Za Zhi* **98**, 191–195 doi:10.3760/cma.j.issn.0376-2491.2018.03.007 (2018).

Montalvo, I. *et al.* Glycated Haemoglobin Is Associated With Poorer Cognitive Performance in Patients With Recent-Onset Psychosis. *Front Psychiatry* **11**, 455 doi:10.3389/fpsyt.2020.00455 (2020).

Nandeesha, H., Keshri, N., Rajappa, M. & Menon, V. Association of hyperglycaemia and hyperlipidaemia with cognitive dysfunction in schizophrenia spectrum disorder. *Arch Physiol Biochem* **129**, 497–504 doi:10.1080/13813455.2020.1839500 (2023).

Pang, L. J. *et al.* [Establishment of diagnostic model for schizophrenia based on neurotrophic factor and other biomarkers]. *Zhonghua Yi Xue Za Zhi* **103**, 1310–1315 doi:10.3760/cma.j.cn112137-20221212-02631 (2023).

Peng, X.-J. *et al.* The Association Between Metabolic Disturbance and Cognitive Impairments in Early-Stage Schizophrenia. *Front Hum Neurosci* **14**, 599720 doi:10.3389/fnhum.2020.599720 (2020).

Salaj, A. et al., The relationship between blood lipid levels, glucose level, thyroid function tests and cognitive functions in first episode schizophrenia patients. *Klinik Psikofarmakoloji Bulteni;*24(0), p. 54. (2014).

Soontornniyomkij, V. *et al.* Clinical Correlates of Insulin Resistance in Chronic Schizophrenia: Relationship to Negative Symptoms. *Front Psychiatry* **10**, 251 doi:10.3389/fpsyt.2019.00251 (2019).

Storch Jakobsen, A. *et al.* Associations between clinical and psychosocial factors and metabolic and cardiovascular risk factors in overweight patients with schizophrenia spectrum disorders - Baseline and two-years findings from the CHANGE trial. *Schizophr Res* **199**, 96–102 doi:10.1016/j.schres.2018.02.047 (2018).

Takayanagi, Y., Cascella, N. G., Sawa, A. & Eaton, W. W. Diabetes is associated with lower global cognitive function in schizophrenia. *Schizophr Res* **142**, 183–187 doi:10.1016/j.schres.2012.08.034 (2012).

Tang, S. X. *et al.* Metabolic disturbances, hemoglobin A1c, and social cognition impairment in Schizophrenia spectrum disorders. *Transl Psychiatry* **12**, 233 doi:10.1038/s41398-022-02002-z (2022).

Tao, Q. *et al.* Insulin Resistance and Oxidative Stress: In Relation to Cognitive Function and Psychopathology in Drug-Naïve, First-Episode Drug-Free Schizophrenia. *Front Psychiatry* **11**, 537280 doi:10.3389/fpsyt.2020.537280 (2020).

Zhang, B. H. *et al.* Gender differences in cognitive deficits in schizophrenia with and without diabetes. *Compr Psychiatry* **63**, 1–9 doi:10.1016/j.comppsych.2015.07.003 (2015).

Zhang, C. *et al.* Metabolic adverse effects of olanzapine on cognitive dysfunction: A possible relationship between BDNF and TNF-alpha. *Psychoneuroendocrinology* **81**, 138–143 doi:10.1016/j.psyneuen.2017.04.014 (2017).

Zhang, X. *et al.* Glucose disturbances, cognitive deficits and white matter abnormalities in first-episode drug-naive schizophrenia. *Mol Psychiatry* **25**, 3220–3230 doi:10.1038/s41380-019-0478-1 (2020).

Yuan, X. et al. Insulin resistance links dysbiosis of gut microbiota with cognitive impairment in first-episode drug-naïve schizophrenia. *Psychoneuroendocrinology* **172**, 107255 (2025).
